# Neonatal resuscitation practices in Italy: a survey of the Italian Society of Neonatology (SIN) and the Union of European Neonatal and Perinatal Societies (UENPS)

**DOI:** 10.1186/s13052-022-01260-3

**Published:** 2022-06-02

**Authors:** Camilla Gizzi, Daniele Trevisanuto, Luigi Gagliardi, Giulia Vertecchi, Stefano Ghirardello, Sandra Di Fabio, Corrado Moretti, Fabio Mosca

**Affiliations:** 1 Department of Pediatrics and Neonatology, Ospedale Sandro Pertini, Rome, Italy; 2 Union of European Neonatal and Perinatal Societies (UENPS), Milan, Italy; 3 Department of Woman’s and Child’s Health, University of Padova, Padova, Italy; 4 Department of Neonatology and Pediatrics, Ospedale Versilia, Viareggio; Azienda USL Toscana Nord Ovest, Pisa, Italy; 5 Neonatal Intensive Care Unit, Fondazione IRCCS Policlinico San Matteo, Pavia, Italy; 6 Department of Pediatrics, Ospedale San Salvatore, L’Aquila, Italy; 7 Department of Pediatrics, Policlinico Umberto I, Sapienza University, Rome, Italy; 8 Department of Pediatrics, Fondazione IRCCS Cà Granda Ospedale Maggiore Policlinico, Milan, Italy; 9 Italian Society of Neonatology (SIN), Milan, Italy

**Keywords:** Italy, Delivery room, Neonatal resuscitation, Survey

## Abstract

**Background:**

Providing appropriate care at birth remains a crucial strategy for reducing neonatal mortality and morbidity. We aimed to evaluate the consistency of practice and the adherence to the international guidelines on neonatal resuscitation in level-I and level-II Italian birth hospitals.

**Methods:**

This was a cross-sectional electronic survey. A 91-item questionnaire focusing on current delivery room practices in neonatal resuscitation was sent to the directors of 418 Italian neonatal facilities.

**Results:**

The response rate was 61.7% (258/418), comprising 95.6% (110/115) from level-II and 49.0% (148/303) from level-I centres. In 2018, approximately 300,000 births occurred at the participating hospitals, with a median of 1664 births/centre in level-II and 737 births/centre in level-I hospitals. Participating level-II hospitals provided nasal-CPAP and/or high-flow nasal cannulae (100%), mechanical ventilation (99.1%), HFOV (71.0%), inhaled nitric oxide (80.0%), therapeutic hypothermia (76.4%), and extracorporeal membrane oxygenation ECMO (8.2%). Nasal-CPAP and/or high-flow nasal cannulae and mechanical ventilation were available in 77.7 and 21.6% of the level-I centres, respectively. Multidisciplinary antenatal counselling was routinely offered to parents at 90.0% (90) of level-II hospitals, and 57.4% (85) of level-I hospitals (*p* < 0.001). Laryngeal masks were available in more than 90% of participating hospitals while an end-tidal CO_2_ detector was available in only 20%. Significant differences between level-II and level-I centres were found in the composition of resuscitation teams for high-risk deliveries, team briefings before resuscitation, providers qualified with full resuscitation skills, self-confidence, and use of sodium bicarbonate.

**Conclusions:**

This survey provides insight into neonatal resuscitation practices in a large sample of Italian hospitals. Overall, adherence to international guidelines on neonatal resuscitation was high, but differences in practice between the participating centres and the guidelines exist. Clinicians and stakeholders should consider this information when allocating resources and planning perinatal programs in Italy.

**Supplementary Information:**

The online version contains supplementary material available at 10.1186/s13052-022-01260-3.

## Introduction

Approximately 5–10% of all newborns need support for transition to initiate breathing and aerate the lungs, while less than 1% require advanced resuscitation at birth, including tracheal intubation, chest compressions, and medications [1–3]. Inadequate cardiorespiratory support at birth may result in pulmonary damage and worsen ongoing hypoxia or ischemia, with the risk of aggravating patient outcomes [2, 3]. To improve resuscitation practices worldwide, the International Liaison Committee on Resuscitation (ILCOR) promulgates and regularly updates through rigorous and continuous review of scientific literature the consensus on both the science of neonatal resuscitation and recommendations for treatment. These updates have formed a basis from which individual countries drew up their own guidelines. Of these, the guidelines drawn up by the American Academy of Pediatrics (AAP)/American Heart Association (AHA) are notable and were adopted by the Italian Society of Neonatology (SIN) and its Task Force on Neonatal Resuscitation since 1994. To improve compliance with the guidelines and to promote adequate knowledge of newborn life support, SIN has invested heavily in neonatal resuscitation training sessions for national instructors, national and on-site neonatal resuscitation courses for practitioners and meetings and congresses on the topic.

Issuing and disseminating recommendations is, however, not sufficient, so we set out to evaluate the degree of uptake of these recommendations. Therefore, the aim of our study was to assess the adherence to Neonatal Resuscitation Guidelines in Italian centres, and to compare the consistency of practice between level-I and level-II centres with the hope that the results of our study would help to shape the strategies for diffusion of the current guidelines according to actual needs.

## Methods

This cross-sectional study was conducted as an electronic, web-based survey involving all Italian birth centres. According to the 2021 Italian standards of perinatal care [4], level-I centres take care of low- and medium-risk pregnancies and assist both healthy newborns and infants with intermediate diseases. Centres with 500–999 births/year can admit infants with GA ≥36 weeks and BW ≥1900 g, while centres with ≥1000 births/year can admit infants with GA ≥34 weeks and BW ≥1750 g, if the weight is adequate for GA (> 10° centile and < 90° centile). However, at the time of the survey, Italian level-I units admitted infants with GA ≥34 weeks, and some of them infants with GA ≥32 weeks. Level-II centres, in addition to the first level of care, are responsible for high-risk pregnancies and assist newborn infants with complex pathologies in neonatal intensive-care beds.

This Italian survey is part of a European survey on delivery room practices endorsed by the Union of European Neonatal and Perinatal Societies (UENPS) and SIN [5]. The study was approved by the Padua Provincial Institutional Review Board and was declared not to be human subject research.

The survey was anonymous and sent to the directors of all Italian birth centres between January and September 2020 by e-mail link (www.surveymonkey.com). A reminder was sent to non-responders every 2 weeks for a maximum of three times; if no answer was received, we contacted the participant by phone. The participant was considered a non-responder if no response was obtained after the phone call.

The survey consisted of a 91-item questionnaire focusing on current Delivery Room (DR) practices of neonatal resuscitation [6]. It was broken down into the following sections: a) epidemiological data, b) perinatal organization, c) equipment, d) procedures, e) ethics, and f) education. The questionnaire was prepared by a committee of experts in neonatal resuscitation and members of the SIN Task Force on Neonatal Resuscitation. The questions included multiple-choice, fill-in, and yes/no questions (the complete questionnaire can be consulted in the supplementary materials).

## Statistical analysis

All returned questionnaires were reviewed by two researchers working separately to avoid duplication of data. Data were examined with descriptive analyses. Categorical data were expressed as numbers and percentages and continuous data as median and interquartile ranges (IQR). In the analysis, level-I and level-II centres were compared using χ^2^ tests. Statistical analysis was performed using Stata 15 Statistical Package (StataCorp. 2017. *Stata Statistical Software: Release 15*. College Station, TX: StataCorp LLC).

## Results

In total, 418 neonatologists and paediatricians identified as directors of birth centres were invited to participate in the survey. The overall response rate to the questionnaire was 61.7% (258/418), 95.6% (110/115) for level-II centres and 49.0% (148/303) for level-I centres. Missing values to individual questions were always < 10%, except in 1 question (see text and tables). Of the participating centres, 49 (11.7%) were academic hospitals. Among these, 43 (39.1%) were level-II centres and 6 (4.0%) were level-I centres.

In 2018, approximately 300,000 births occurred at the participating hospitals (about 70% of all Italian births). The median of births/centre was 1664 (IQR: 1250-2391) at level-II and 737 (IQR: 525–1035) at level-I centres. The main results of the survey are reported in Tables 1, 2, 3, 4 and 5, broken down by questionnaire section. Participating level-II hospitals were able to provide nasal-CPAP and/or high-flow nasal cannulae (100%), mechanical ventilation (99.1%), HFOV (71.0%), inhaled nitric oxide (80.0%), therapeutic hypothermia (76.4%), and ECMO (8.2%). Nasal-CPAP and/or high-flow nasal cannulae and mechanical ventilation were available in 77.7 and 21.6% of the level-I centres, respectively. In 74.5% of the level-II centres, the lowest GA of assisted infants was 22–23 weeks, while among level-I centres the lowest GA of assisted infants was 32 weeks in 20.6% and 34 weeks in 53.9%.

**Table 1:** Before Birth

BEFORE BIRTH			
	Level II (n.110)	Level I (n.148)	***p***
**At your hospital, is the antenatal counseling with parents routinely performed before the delivery of a very preterm infant or an infant with expected problems?**			
• yes	99 (90)	85 (57.4)	**< 0.001**
• no	11 (10)	63 (42.6)	
**Who are the specialists involved in antenatal counseling?**			
• Pediatrician/Neonatologist	99 (90.0)	82 (55.4)	**< 0.001**
• Obstetrician	90 (81.8)	72 (48.6)	**< 0.001**
• Anesthesiologist	16 (14.5)	25 (16.8)	NS
• Other	31 (28.2)	7 (4.7)	0.05
**How is Resuscitation Team for low-risk delivery composed?**			
• Pediatrician/Neonatologist	84 (76.4)	112 (75.6)	NS
• Obstetrician	21 (19.1)	34 (22.9)	NS
• Anesthesiologist	2 (1.8)	19 (12.8)	**0.001**
• Midwife	45 (40.9)	71 (47.9)	NS
• Nurse	64 (58.2)	106 (71.6)	**0.033**
• Other	10 (9.1)	1 (0.7)	NS
**How is Resuscitation Team for high-risk delivery composed?**			
• Pediatrician/Neonatologist	108 (98.2)	145 (97.9)	NS
• Obstetrician	25 (22.7)	46 (31.0)	NS
• Anesthesiologist	30 (27.3)	117 (79.0)	**< 0.001**
• Midwife	33 (30.0)	60 (40.5)	0.08
• Nurse	100 (90.9)	121 (81.7)	**0.038**
• Other	12 (10.9)	9 (6.1)	NS
**Which provider is qualified for full resuscitation skills among your resuscitation team members (endotracheal intubation, chest compressions, emergency vascular access and administration of medication)?**			
• Pediatrician/Neonatologist	89 (80.9)	22 (13.5)	**< 0.001**
• Anesthesiologist	5 (4.5)	24 (16.2)	**0.007**
• Pediatrician/Neonatologist and Anesthesiologist	25 (22.7)	109 (73.6)	**< 0.001**
**Do you usually have a team briefing before resuscitation?**			
• yes	70 (63.6)	64 (43.2)	**0.001**
• no	40 (36.4)	84 (56.8)	

Data are expressed as number (%)

**Table 2:** Umbilical cord management

UMBILICAL CORD MANAGEMENT			
	Level II (n.110)	Level I (n.148)	***p***
**Umbilical cord management in term vaginal delivery**			NS
• Delayed cord clamping	73 (66.3)	84 (56.8)	
• Physiologically-based cord clamping^a^	31 (28.2)	49 (33.1)	
• Immediate cord clamping	6 (5.4)	8 (5.4)	
• Umbilical cord milking	0	1 (0.7)	
• NA	0	6 (4.1)	
**Umbilical cord management in term elective Cesarean Section**			**0.008**
• Delayed cord clamping	61 (55.4)	52 (35.1)	
• Physiologically-based cord clamping	13 (11.8)	26 (17.6)	
• Immediate cord clamping	30 (27.3)	56 (37.8)	
• Umbilical cord milking	6 (5.4)	8 (5.4)	
• NA	0	6 (4.1)	

Data are expressed as number (%)

^a^ deferring umbilical cord clamping until after lung aeration

**Table 3:** Temperature

TEMPERATURE			
	Level I (n.110)	Level II (n.148)	***p***
**Which is your DR temperature?**			
• degrees	24 (23–25)	24 (23–25)	NS
• I don’t know	21 (19.1)	48 (32.4)	**0.017**
**Which is your Operating room temperature?**			
• degrees (median, 25–75 centile)	23 (20–24)	22(21–24)	NS
• I don’t know	26 (23.6)	56 (37.8)	**0.015**
**At your Institution, when is “passive cooling” (i.e. switch off of the radiant warmer) started for newborn infants who are considered at risk of hypoxic-ischemic encephalopathy?**			**0.009**
• 1–2 h	2 (1.8)	13 (9.1)	
• 2–4 h	0	3 (2.1)	
• 4–6 h	2 (1.8)	3 (2.1)	
• We do not have a specific time	7 (6.4)	15 (10.5)	
• Within 1 h	99 (90.0)	108 (76.0)	
• Missing	0	6 (4.0)	

Data are expressed as number (%) or median (IQR)

**Table 4:** Airways, ventilation, circulation and medications

	Level II (n.110)	Level I (n.148)	***p***
**AIRWAYS**			
**How is a non-vigorous newborn infant born through meconium-stained amniotic fluid managed in your DR?**			**0.005**
• Routine tracheal suction	20 (18.2)	10 (7.0)	
• Starting PPV	84 (76.3)	118 (83.1)	
• Oro-nasopharyngeal suction on the perineum	6 (5.45)	14 (9.9)	
• Missing	0	6 (4.1)	
**VENTILATION**			
**DR Equipment**			
• Air/oxygen blender	108 (98.2)	138 (97.9)	NS
• Pulse oxymeter	108 (98.2)	137 (97.2)	NS
**Initial FiO**_**2**_ **if PPV is needed in GA ≥ 35 wks**			NS
• 0.21	98 (89.1)	127 (91.3)	
• 0.25	0	1 (0.7)	
• 0.28	0	1 (0.7)	
• 0.3	10 (9.1)	6 (4.3)	
• 0.35	0	1 (0.7)	
• 0.4	1 (0.9)	2 (1.4)	
• 1	1 (0.9)	1 (0.7)	
• Missing	0	9 (6.1)	
**Initial FiO**_**2**_ **if PPV is needed in GA < 35 wks**			NS
• 0.21	35 (31.8)	47 (34.06)	
• 0.25	8 (7.3)	4 (2.9)	
• 0.3	62 (56.4)	80 (57.9)	
• 0.4	2 (1.8)	2 (1.4)	
• 0.5	0	2 (1.4)	
• 0.6	0	1 (0.7)	
• 1	1 (0.9)	2 (1.4)	
• Missing	2 (1.8)	10 (6.7)	
**In your delivery room, PPV at birth is routinely administered with:**			NS
• Flow-inflating bag	4 (3.64)	7 (4.9)	
• Neonatal Mechanical ventilator	2 (1.82)	1 (0.7)	
• Other	3	0	
• Self-inflating bag	1 (0.91)	3 (2.1)	
• T-piece device (Neopuff)	101 (91.8)	129 (91.5)	
**Which ventilatory interface is routinely used as the first choice in your center?**			**0.067**
• Facial mask	92 (83.6)	131 (92.9)	
• Nasopharyngeal prongs	3 (2.7)	1 (0.7)	
• Short binasal prongs	13 (11.8)	8 (5.4)	
• Missing	2 (1.8)	8 (5.4)	
**Is Laryngeal Mask part of the equipment in your DR?**			NS
• Yes	99 (90.0)	130 (92.2)	
• No	11 (10.0)	11 (7.8)	
• Missing	0	7 (4.7)	
**What are the CPAP levels routinely administered in late preterm and term infants (i.e. GA ≥ 33 weeks) In your DR?**			**< 0.001**
• ≤4	8 (7.3)	26 (17.6)	
• 5	64 (58.2)	80 (54.1)	
• 6	32 (29.1)	12 (8.1)	
• 7	3 (2.7)	1 (0.7)	
• Missing	3 (2.7)	29 (19.6)	
**What are the PEEP levels routinely administered to start PPV in late preterm and term infants (i.e. GA ≥ 33 weeks) In your DR?**			**< 0.001**
• ≤4	12 (10.9)	30 (20.3)	
• 5	67 (60.9)	93 (62.8)	
• 6	30 (27.3)	8 (5.4)	
• 8	0	2 (1.4)	
• missing	1 (0.9)	15 (10.1)	
**What are the PIP levels routinely administered to start PPV in late preterm and term infants (i.e. GA ≥ 33 weeks) in your DR?**			**0.012**
• < 20	17 (15.4)	11 (7.4)	
• 20	45 (40.9)	69 (46.6)	
• 25	33 (30.0)	35 (23.7)	
• 30	1 (0.9)	5 (3.4)	
• Other	10 (9.1)	8 (5.4)	
• Missing	4 (3.6)	20(13.5)	
**Are heated, humidified gases available for PPV/respiratory support?**			NS
• Yes	65 (59.1)	88 (59.5)	
• No	45 (40.9)	60 (40.5)	
**In the case of endotracheal intubation, is an end-tidal CO**_**2**_ **detector used to confirm the correct placement of the tracheal tube?**			NS
• Yes	21 (19.1)	31 (20.9)	
• No	89 (80.9)	117 (79.0)	
**How skilled is your team on intubation?**			**0.000**
• Excellent	62 (56.4)	10 (7.1)	
• Good	41 (37.3)	49 (34.7)	
• I don’t know/NA	2 (1.82)	9 (6.1)	
• Insufficient	0	22 (15.6)	
• Sufficient	5 (4.55)	58 (41.1)	
**CIRCULATION**			
**How do healthcare givers at your institution detect and monitor the newborn’s heart rate in neonates needing resuscitation?**			
• Palpation of the umbilical cord	11 (10.0)	26 (17.6)	0.086
• Palpation of peripheral pulses	2 (1.8)	2 (1.3)	NS
• Stethoscope	84 (76.4)	102 (68.9)	NS
• Three lead ECG monitor	46 (41.8)	46 (31.1)	0.075
• Pulse oximeter	74 (62.3)	103 (69.6)	NS
**MEDICATIONS**			
**Do you use Sodium bicarbonate in the DR?**			**0.003**
• No	102 (92.7)	114 (82.1)	
• Sometimes	5 (4.5)	25 (18.0)	
• Missing	3 (2.7)	9 (6.1)	

Data are expressed as number (%)

**Table 5:** Ethics and education

	Level II (n.110)	Level I (n.148)	***p***
**ETHICS**			
**In severely asphyxiated infants is there a time-limit before stopping full resuscitation in your hospital?**			**0.045**
• I don’t know/NA	5 (4.5)	16 (10.8)	
• No	42 (38.2)	64 (43.2)	
• Yes	63 (57.3)	68 (46.0)	
**Does parental opinion influence intervention in your center?**			0.088
• A little bit	40 (36.4)	46 (31.1)	
• I don’t know/NA	3 (2.7)	13 (8.8)	
• No, at all	31 (28.2)	49 (33.1)	
• Yes, enough	36 (32.7)	40 (27.0)	
**EDUCATION**			
**Are courses on neonatal resuscitation routinely held at your hospital?**			**0.021**
• Yes	96 (87.3)	114 (77.0)	
• No	14 (12.7)	26 (17.6)	
• Missing	0	8 (5.4)	
**How often are neonatal resuscitation teams retrained?**			NS
• 12–24 months	59 (53.6)	69 (46.6)	
• 6–12 months	31 (28.2)	40 (27.0)	
• < 6 months	4 (3.6)	7 (4.7)	
• > 24 months	16 (14.5)	24 (16.2)	
• NA	0	8 (5.4)	

Data are expressed as number (%)

Multidisciplinary antenatal counselling was routinely offered to parents at 90.0% (90) of level-II hospitals, and 57.4% (85) of level-I hospitals (*p* < 0.001). A neonatologist or paediatrician was required to attend all deliveries in about 60% of centres at each level.

Significant differences between level-II and level-I centres were found mainly in antenatal counselling, composition of the resuscitation team for high-risk deliveries, team briefings before resuscitation, providers qualified with full resuscitation skills and role of the anaesthesiologist (Table 1), lack of awareness about temperature (Table 3), routine tracheal suction for *non*-*vigorous* neonates born through *meconium*-*stained* amniotic fluid, self-confidence, sodium bicarbonate use (Table 4) and frequency of neonatal resuscitation courses (Table 5).

## Discussion

Overall, our survey documents good compliance with international guidelines on neonatal resuscitation in most Italian level-II and level-I centres. However, as expected, there are some differences, mostly in perinatal care.

### Before birth

A lower number of births/centre and less complexity in the assisted cases account for the differences in how the levels approach the period ‘before birth’. Indeed, before delivering a very preterm infant or an infant with anticipated problems, counselling with parents is routinely performed more often at level-II centres, which expect to assist the most vulnerable newborn infants. For the same reason, paediatricians and neonatologists working in level-I hospitals are less trained for emergencies, so an anaesthesiologist is more frequently needed on the resuscitation team.

### Umbilical cord management

Delaying cord clamping (DCC) at birth is associated with increased haemoglobin levels and better iron stores in newborn infants [7]; it favours the cardiovascular transition that occurs in the first minutes of life [8, 9] and decreases mortality among infants with GA < 37 weeks [10]. Delayed or physiologically based cord clamping (i.e. aerating the lung before removing placental support) is provided to most vaginally delivered term infants, suggesting that these interventions were introduced in Italy after umbilical cord management recommendations were disseminated [11]. However, in term elective caesarean section, delayed strategies decrease to 67.2% in level-II and 52.7% in level-I hospitals, indicating the need to implement umbilical cord management for these otherwise healthy newborn infants. As suggested by recent evidence, delaying cord clamping does not affect maternal blood losses [12, 13].

### Temperature

A high incidence of postnatal hypothermia has been reported in high- and low-resource countries. It remains an independent predictor of neonatal morbidity and mortality, especially in very preterm infants in all settings. The International Guidelines suggest that the temperature of newly born infants should be maintained between 36.5 and 37.5 °C after birth through admission and stabilization [2, 3]. Effective interventions to achieve this may include environmental temperature 23–25 °C, use of radiant warmers, exothermic mattresses, woollen or plastic caps, plastic wraps, humidified and heated gases [14]. According to our survey, neonatologists in level-I centres are less aware than those in level-II of the importance of keeping the delivery room and operating room temperatures within the suggested ranges as part of the effective interventions to prevent thermal losses at birth.

It is well known that therapeutic hypothermia should be started within the first 6 h of life for newborn infants at risk of hypoxic-ischemic encephalopathy [15]. We observed good compliance with this practice in all Italian birth centres, in most of which ‘passive cooling’ is started within 1 h of life.

### Airways, ventilation, circulation and medications

Since 2015, the International Guidelines have recommended against routine endotracheal suctioning of meconium-stained non-vigorous newborns, instead suggesting resuscitation with positive pressure ventilation. Our survey showed that both level-II and level-I centres had changed their practice accordingly; however, in level-II centres, routine tracheal suction is still performed more often, reflecting a greater confidence with the intubation manoeuvre.

Effective ventilation is considered the most critical intervention for successful delivery room resuscitation [16]. Most perinatal management of this aspect is comparable between birth centres, especially in the use of a ‘gentle’ approach to ventilation. In both level-II and level-I hospitals, a T-piece is preferred to a self-inflating bag to administer positive pressure ventilation (PPV), predominantly using a face mask as the first interface. However, in 12% of level-II centres, short binasal prongs are the first choice. This solution seems to offer some advantages over face masks in terms of reducing intubation in the delivery room [17–19].

An air-oxygen blender and a pulse oximeter to guide oxygen titration are available in almost all Italian delivery rooms. Moreover, as recommended by the International Guidelines, a laryngeal mask is part of the equipment in more than 90% of the participating hospitals, to be used in the event of failure of face mask ventilation or intubation [2, 3]. On the other hand, an end-tidal CO_2_ detector, which is considered the most reliable tool to identify the correct placement of the endotracheal tube [20], is only available in about 20% of the responding birth centres. This device could potentially decrease the number of intubation attempts and improve outcomes [21, 22]. Among technical aspects, fewer level-I neonatal resuscitation teams self-evaluated as excellent or good at performing the endotracheal intubation manoeuvre. By contrast, this is a well-acquired skill by level-II teams, who are expected to assist the most vulnerable newborn infants routinely.

Since 2015, electrocardiography is recognized as an important adjunct for babies requiring resuscitation. Nevertheless, a 3-lead ECG Monitor is only available in a quarter of responding centres, showing limited adherence to the latest version of the guidelines, which recommend its use in infants needing advanced resuscitation [2, 3].

Finally, although sodium bicarbonate is no longer considered helpful during acute resuscitation [23], it is still used on occasion, especially in level-I centres.

### Ethics and education

Our survey shows that awareness of ethical issues should be reinforced in Italy. Indeed, a difficult decision like the time-limit before stopping full resuscitation in severely asphyxiated infants is not supported by shared guidelines in about 50% of responding hospitals. Moreover, parents are involved in the decision-making process of resuscitation in only one third of Italian centres. These findings are in line with previous European studies [24, 25]. Ethics remains a delicate field that needs further research to help neonatal staff and parents deal with difficult situations.

Courses on neonatal resuscitation are routinely held in more than 80% of Italian birth centres, and most of these follow the American or European guidelines. Frequency of retraining is also optimal in about 80% of centres, according to the 2020 recommendations, suggesting that among participants who have been trained in neonatal resuscitation, individual or team booster-training should occur more frequently than every 2 years in order to support retention of knowledge, skills, and behaviour.

### Strengths and limitations

The strengths of the present study include the structured questionnaire prepared by a group of experts; the assessment of several areas of neonatal resuscitation; the high representativity of the sample, which accounts for about 70% of all Italian deliveries in 2018; and finally, the use of an online survey.

This study has some limitations, starting from the consideration that conducting surveys in an online format runs the risk of selection bias [26]. The response rate is at the same time a strength for level-II hospitals and a limitation for those at level-I. The limited response rate (49.0%) of level-I centres may restrict the generalizability of our findings. Finally, as only the directors of neonatal wards were involved, the results may mirror the opinions of this very restricted group of clinicians; nevertheless, the questionnaire was structured to limit this risk.

## Conclusions

This survey provides insight into neonatal resuscitation practices in a large sample of Italian hospitals. Overall, adherence to the International Guidelines on neonatal resuscitation was high, but we also saw some divergences in practice, ethical choices, and training among the participating centres. Clinicians and stakeholders should consider this information when allocating resources and planning Italian perinatal programmes. The areas of the current guidelines that require further implementation include aspects of perinatal care, respiratory support, ethics and training. This goal can be achieved by defining new educational strategies, including quality improvement projects, simulation-based training and interventions in communication technology.

## Supplementary Information

**Additional file 1: Supplementary file 1.** PDF Questionnaire, complete survey questionnaire.

**Additional file 2: Supplementary file 2.** List of participating centres, complete list of participating centres.

## Data Availability

The datasets used and/or analysed during the current study are available from the corresponding author on reasonable request.

## Abbreviations

SIN: Società Italiana di Neonatologia (Italian Society of Neonatology)
UENPS: Union of European Neonatal and Perinatal Societies
HFOV: High-frequency oscillatory ventilation
ECMO: Extracorporeal membrane oxygenation
ILCOR: International Liaison Committee on Resuscitation
AAP: American Academy of Pediatrics
AHA: American Heart Association
GA: Gestational Age
BW: Birth Weight
IQR: Interquartile Range
PPV: Positive pressure ventilation
