# Supplementary material for: Neonatal resuscitation practices in Italy: a survey of the Italian Society of Neonatology (SIN) and the Union of European Neonatal and Perinatal Societies (UENPS)
Source: Ital J Pediatr. 2022 Jun 2;48:81. doi: 10.1186/s13052-022-01260-3 (PMC9164545; doi:10.1186/s13052-022-01260-3)
Supplement: Supplementary file 1 — Additional file 1: Supplementary file 1. PDF Questionnaire, complete survey questionnaire. [file 13052_2022_1260_MOESM1_ESM.pdf]

A Survey on Delivery Room Resuscitation in Europe

Approximately 5 to 10% of the newly born population requires some degree of active resuscitation at birth (for example, stimulation to breathe) and about 1 to 10% of these infants are reported to receive assisted ventilation. Neonatal resuscitation is the most frequent resuscitation intervention provided in hospitals.

The purpose of this survey is to assess the current situation regarding the neonatal resuscitation practices in a large representative sample of delivery rooms in Europe. The results of this survey may allow knowing the common points, the differences and trends of European neonatologists/pediatricians and physicians involved in this particular area of their activities and could provide a starting point for creating an European collaborative network.

The questionnaire was designed following the classic stages of neonatal resuscitation, including also organizational (personnel and equipment) and epidemiological aspects. It is addressed to levels of perinatal care <u>with at least 1.000 deliveries per year.</u>

We ask you to fill the questionnaire in all its parts, according to the level of assistance of your unit.

Time needed to complete the questionnaire is <u>about 30 minutes</u>.

Thanks for your cooperation and availability.

Best regards.

Daniele Trevisanuto, Camilla Gizzi, Luigi Gagliardi, Stefano Ghirardello, Sandra Di Fabio, Fabio Mosca* and Corrado Moretti$

***on behalf of the Italian Society of Neonatology,**

$ on behalf of UENPS

A Survey on Delivery Room Resuscitation in Europe

General Information

The data provided by each hospital will be covered under the privacy policy of UENPS Society. It will be used to draw up results, and <u>then it will be anonymised before publication.</u>

You can change your answers on any survey page until you complete the survey and click the DONE button.

* 1. Country

[ ]

* 2. Hospital

Name

Address

City/Town

ZIP/Postal Code

Phone Number

* 3. Academic Hospital

- Yes
- No

* 4. Contact Person

Name

Email Address

Phone Number

* 5. Lowest GA of routinely assisted infants in your Delivery Room

* 6. Available Facilities at your Hospital

- NICU
- Nasal-CPAP/HFNC
- Mechanical Ventilation
- High Frequency Oscillatory Ventilation
- Other (please specify)
- Nitric Oxide
- Therapeutic Hypothermia
- ECMO

A Survey on Delivery Room Resuscitation in Europe

Before Birth

* 7. At your hospital is the antenatal counseling with parents routinely performed before the delivery of a very preterm infant or of an infant with expected problems?

- Yes
- No

* 8. If yes, who are the specialists involved in the antenatal counseling?

- [ ] Pediatricians/Neonatologists
- [ ] Obstetricians
- [ ] Anesthesiologists
- [ ] If other (please specify)

* 9. Do you have a check-list for preparing material and equipment in your delivery room?

- ( ) Yes
- ( ) No

* 10. Who is responsible for preparing the material and the equipment?

- [ ] Paediatrician/Neonatologist
- [ ] Obstetrician
- [ ] Anesthesiologist
- [ ] Midwife
- [ ] Nurse
- [ ] If other (please specify)

* 11. How is your Neonatal Resuscitation Team routinely composed **for a low-risk delivery**?

- [ ] Paediatrician/Neonatologist
- [ ] Obstetrician
- [ ] Anesthesiologist
- [ ] Midwife
- [ ] Nurse
- [ ] If other (please specify)

* 12. How is your Neonatal Resuscitation Team routinely composed **for a high-risk delivery**?

- [ ] Paediatrician/Neonatologist
- [ ] Obstetrician
- [ ] Anesthesiologist
- [ ] Midwife
- [ ] Nurse
- [ ] If other (please specify)

* 13. Among your resuscitation team members, which provider is qualified for full resuscitation skills, including endotracheal intubation, chest compressions, emergency vascular access and administration of medication?

- [ ] Paediatrician/Neonatologist
- [ ] Anesthesiologist
- [ ] Pediatrician/Neonatologist and Anesthesiologist
- [ ] If other (please specify)

* 14. Do you usually have a team briefing before resuscitation?

- ( ) Yes
- ( ) No

* 15. How many deliveries took place in your hospital in 2018?

* 16. In your hospital, is the resuscitation team present at every delivery?

- ( ) Yes
- ( ) No

A Survey on Delivery Room Resuscitation in Europe

Umbilical Cord Management in your DR

* 17. How is the umbilical cord managed in **vaginally-delivered term** newborns?

- Immediate CordClamping (ICC)
- Physiologically-Based Cord Clamping (PBCC)
- Milking
- Delayed Cord Clamping (DCC): after how many seconds? (select this option to specify)

* 18. How is the umbilical cord managed in **elective cesarean-delivered term** newborns?

- ICC
- PBCC
- Milking
- DCC: after how many seconds? (please specify)

* 19. How is the umbilical cord managed in **emergency cesarean-delivered term** newborns?

- ICC
- PBCC
- Milking
- DCC: after how many secconds? (please specify)

* 20. How is the umbilical cord managed in **vaginally-delivered late preterm** (i.e GA 33-36 weeks) newborns?

- ICC
- PBCC
- Milking
- DCC: after how many seconds? (please specify)

* 21. How is the umbilical cord managed in **cesarean-delivered late preterm** (i.e GA 33-36 weeks) newborns?

- ICC
- PBCC
- Milking
- DCC: after how many seconds? (please specify)

* 22. In case of PBCC, which parameters are considered in your DR before clamping?

* 23. Who decides the management of cord clamping in extremely preterm deliveries?

* 24. In which situations is umbilical cord milking performed in your DR?

- [ ] In all preterm deliveries
- [ ] In cesarean-delivered term newborns
- [ ] When immediate resuscitation is required
- [ ] In extremely preterm newborns
- [ ] You never perform umbilical cord milking

* 25. Is full-resuscitation with intact umbilical cord on a fully-equipped bedside trolley possible in your DR?

- ( ) Yes
- ( ) No

* 26. When is arterial cord blood gas analysis performed in your DR?

- ( ) Routinely (in all deliveries)
- ( ) In asphyxiated infants
- ( ) Never
- ( ) If other (please specify)

* 27. In your DR, if arterial cord blood gas analysis is deemed necessary immediately after birth and if neonatal clinical conditions at birth are reassuring, is a cord blood gas analysis performed on unclamped cord and is DCC allowed?

- ( ) Yes
- ( ) No

Temperature

* 28. What is the temperature of your delivery room?

- I don't know
- C° (please specify)

* 29. What is the temperature of your operating room?

- I don't know
- C° (please specify)

* 30. At your Institution, when is “passive cooling” (i.e. switch off the radiant warmer) started for newborn infants that are considered at risk of hypoxic-ischemic encephalopathy?

- Within 1 hour
- 1-2 hours
- 2-4 hours
- 4-6 hours
- We do not have a specific time

A Survey on Delivery Room Resuscitation in Europe

Airways

* 31. How is a non-vigorous newborn infant born through meconium-stained amniotic fluid managed in your DR?

- Suctioning of the oro- and nasopharynx on the perineum before delivery of the shoulders
- Routine tracheal intubation of the trachea and suctioning of meconium from beneath the glottis
- Starting PPV after removing secretions, if needed

A Survey on Delivery Room Resuscitation in Europe

Ventilation

* 32. Is your DR equipped with an air/oxygen blender?

- Yes
- No

* 33. Is your DR equipped with a pulse-oximeter to titrate O_2 administration?

- Yes
- No

* 34. If a ≥ 35 weeks newborn infant needs positive pressure ventilation (PPV) immediately after birth, what initial FiO_2 is used in your DR?

* 35. If a preterm infant <35 weeks needs positive pressure ventilation (PPV) immediately after birth, what initial FiO_2 is you used in your DR?

* 36. In your delivery room, PPV at birth is routinely administered with:

- Self-inflating bag
- Flow-inflating (anesthesia) bag
- T-piece device (Neopuff)
- Neonatal mechanical ventilator
- If other (please specify)

* 37. Which ventilatory interface is routinely used as the first choice in your centre?

* 38. Is a laryngeal mask part of the equipment in your DR?

- Yes
- No

* 39. How does your team consider, the laryngeal mask? A device that is…

- Useless
- Helpful to start ventilation as a first choice in all depressed neonates
- I don't know
- Helpful in some situations (please specify)

* 40. How many neonates were resuscitated by using a laryngeal mask in your centre during the last year?

- No-patient
- 1-5 patients
- 6-10 patients
- >10 patients
- I don't know

* 41. What sizes are the laryngeal mask available in your delivery room?

- 0.5
- 1.0
- 1.5
- 2.0
- I don't know

* 42. Would a smaller size of the laryngeal mask (for preterm infants) be helpful in the equipment of your delivery room?

- Yes
- No
- I don't know

* 43. How skilled is your team on the use of facial mask?

Excellent	Good	Sufficient	Insufficient	I don't know
○	○	○	○	○

* 44. How skilled is your team on the use of laryngeal mask?

Excellent	Good	Sufficient	Insufficient	I don't know
○	○	○	○	○

* 45. How skilled is your team on intubation?

Excellent	Good	Sufficient	Insufficient	I don't know
○	○	○	○	○

* 46. Which interface/interfaces is/are most often used for non-invasive respiratory support in your delivery room?

- [ ] Facial mask
- [ ] Short binasal prongs
- [ ] Nasopharyngeal prongs
- [ ] Single nasopharyngeal prong (cut ETT positioned in nasopharynx)
- [ ] If other (please specify)

* 47. In your DR, are heated and humidified gases available for PPV/respiratory support?

- ( ) Yes
- ( ) No

* 48. In the case of endotracheal intubation, is an end-tidal CO_2 detector used to confirm the correct placement of the tracheal tube?

- ( ) Yes
- ( ) No

A Survey on Delivery Room Resuscitation in Europe

* 49. If yes, which type of end-tidal CO_2 detector do you use?

- [ ] Colorimetric
- [ ] Numerical

* 50. In your DR, what are the CPAP levels routinely administered in late preterm and term infants (i.e. GA ≥33 weeks)?

Please specify cmH_2O

* 51. In your DR, what are the PEEP levels routinely administered to start PPV in late preterm and term infants (i.e. GA ≥ 33 weeks)?

Please specify cmH_2O

* 52. In your DR, what are the PIP levels routinely administered to start PPV in late preterm and term infants (i.e. GA ≥ 33 weeks) ?

Please specify cmH_2O

A Survey on Delivery Room Resuscitation in Europe

Circulation

* 53. How do healthcare givers at your institution detect and monitor the newborn’s heart rate in neonates needing resuscitation?

- [ ] Palpation of the umbilical cord
- [ ] Palpation of peripheral pulses
- [ ] Stethoscope
- [ ] Three lead ECG Monitor
- [ ] Pulse oximeter
- [ ] I don't know

A Survey on Delivery Room Resuscitation in Europe

Ethics

* 54. In your hospital, is there a time-limit before you stop full resuscitation in severely asphyxiated infants?

- ( ) I don't know
- ( ) no
- ( ) If yes, please specify

* 55. Does parental opinion influence your intervention in your centre?

No, at all	A little bit	Yes, enough	I don't know
( )	( )	( )	( )

A Survey on Delivery Room Resuscitation in Europe

Documentation (after resuscitation)

* 56. In the case of birth asphyxia, how do you consider the quality of documentation provided by your staff?

Excellent	Good	Sufficient	Insufficient	I don't know
( )	( )	( )	( )	( )

* 57. In your center, do you have a predefined format for helping caregivers to document neonatal resuscitation in asphyxiated infants?

- Yes
- No

* 58. Do you think that the availability of a form (indicating procedures and times) could be useful to your team for improving the quality of documentation on the resuscitation of asphyxiated infants?

- Yes
- No

A Survey on Delivery Room Resuscitation in Europe

Education

* 59. Are courses on neonatal resuscitation routinely held at your hospital?

- Yes
- No

A Survey on Delivery Room Resuscitation in Europe

* 60. If yes, which algorithm for Neonatal Resuscitation does your institution follow?

- European Resuscitation Council (ERC)
- American Academy of Pediatrics (AAP)
- National Guidelines
- I don’t know
- If other (please specify)

A Survey on Delivery Room Resuscitation in Europe

* 61. Please specify which National Guidelines

* 62. How often are neonatal resuscitation teams retrained?

- < 6 months
- 6-12 months
- 12-24 months
- > 24 months

A Survey on Delivery Room Resuscitation in Europe

Additional Questions for Centres who care for infants with GA <33 weeks

The following questions are intended mostly for Vermont-Oxford Network (VON) members

63. How many infants with BW<1500 g were admitted to your NICU in 2018?

Inborn:

Outborn:

64. How many infants with BW<1500 g received PPV in your delivery room in 2018?

65. How many infants with BW <1500 g were intubated in your delivery room in 2018?

66. How many infants with BW <1500 g needed chest compressions in your delivery room in 2018?

67. How many infants with BW <1500 g received medications in your delivery room in 2018?

A Survey on Delivery Room Resuscitation in Europe

Umbilical Cord Management in your DR for preterm infants

* 68. How is the umbilical cord managed in **vaginally delivered 29-32 week preterm** newborns?

- ICC
- PBCC
- Milking
- I dont know
- DCC: after how many seconds? (please specify)

* 69. How is the umbilical cord managed in **cesarean delivered 29-32 week preterm** newborns?

- ICC
- PBCC
- Milking
- I dont know
- DCC: after how many seconds? (please specify)

* 70. How is the umbilical cord managed in **vaginally delivered <29 week preterm** newborns?

- ICC
- PBCC
- Milking
- I dont know
- DCC: after how many seconds? (please specify)

* 71. How is the umbilical cord managed in **cesarean delivered <29 week preterm** newborns?

- ICC
- PBCC
- Milking
- I dont know
- DCC: after how many seconds? (please specify)

Temperature for preterm infants

* 72. Which strategies do you use in your DR to keep a preterm infant <32 weeks warm?
Check all strategies routinely adopted in your institution

- ☐ Increasing the DR temperature before birth
- ☐ Preheating the radiant warmer before the time of birth
- ☐ Using a servo-controlled temperature probe
- ☐ Pre-warmed towels
- ☐ Polyethylene plastic bag or wrap
- ☐ Hat
- ☐ Thermal mattress
- ☐ Heated und humidified gases for ventilation/respiratory support

Ventilation for preterm infants

* 73. If a preterm infant <29 weeks needs positive pressure ventilation (PPV) immediately after birth, what initial FiO_2 is used in your DR?

____

* 74. In your DR, is sustained lung inflation applied at birth before PPV?

- ○ Yes, routinely
- ○ Yes, occasionally
- ○ No

A Survey on Delivery Room Resuscitation in Europe

* 75. If yes, which parameters do you administer?

Seconds	
Pressure (cmH2O)	
FiO2	
If needed, repeated max	

* 76. In your DR, what are the CPAP levels routinely administered in preterm infants (i.e. GA <33 weeks)?
Please specify cmH$_2$O

* 77. In your DR, what are the PEEP levels routinely administered to start PPV in preterm infants (i.e. GA <33 weeks) ?
Please specify cmH$_2$O

* 78. In your DR, what are the PIP levels routinely administered to start PPV in preterm infants (i.e. GA <33 weeks) ?
Please specify cmH$_2$O

* 79. In your DR, is respiratory function monitoring used during resuscitation (i.e., pressure, flow and volume traces, capnography, etc.)?

- Yes
- No

* 80. How are infants usually ventilated during transport from the DR to the NICU?

- Manual ventilation
- Mechanical ventilator
- Spontaneous breathing, due to the short distance
- CPAP system (please specify)

* 81. Which interface/interfaces is/are most often used for non-invasive respiratory support during the transfer of the baby to the NICU?

- [ ] Facial mask
- [ ] Short binasal prongs
- [ ] Nasopharyngeal prongs
- [ ] Single nasopharyngeal prongs (cut ETT positioned in nasopharynx)
- [ ] If other (please specify)

A Survey on Delivery Room Resuscitation in Europe

Medications for preterm infants

* 82. Do you use caffeine in your delivery room?

- ( ) Yes
- ( ) No

A Survey on Delivery Room Resuscitation in Europe

* 83. If yes, in which infants?

Weeks gestation (please specify)

* 84. If yes, in what dose (caffeine base)?

mg/kg (please specify)

A Survey on Delivery Room Resuscitation in Europe

* 85. Do you use surfactant in your delivery room?

- ( ) Yes
- ( ) No

A Survey on Delivery Room Resuscitation in Europe

* 86. If yes, in which infants?

All neonates with less than ______weeks (prophylaxis)

All neonates with GA less than ______weeks who need intubation for primary resuscitation

If other (please specify)

* 87. Do you use surfactant in DR for selected infants according to GA and FiO_2?

- Yes
- No

A Survey on Delivery Room Resuscitation in Europe

* 88. If yes, please specify:

Below which GA

Above which FiO2

A Survey on Delivery Room Resuscitation in Europe

* 89. If you use surfactant in your DR, which approach/approaches is/are usually used in your hospital?

- LISA
- INSURE
- Oro-pharyngeal administration
- ETT left in place

A Survey on Delivery Room Resuscitation in Europe

Ethics for preterm infants

* 90. In your hospital, is there a gestational age limit for initiating full resuscitation at birth?

- No
- I don't Know
- If yes, please specify

A Survey on Delivery Room Resuscitation in Europe

Medications

IMPORTANT: Please do not forget to click on both DONE buttons at the end of the Survey!

* 91. Do you use sodium bicarbonate in the Delivery Room?

- No
- If yes, in which infants? (please specify)
