# Supplementary material for: Neonatal resuscitation practices in Italy: a survey of the Italian Society of Neonatology (SIN) and the Union of European Neonatal and Perinatal Societies (UENPS)
Source: Ital J Pediatr. 2022 Jun 2;48:81. doi: 10.1186/s13052-022-01260-3 (PMC9164545; doi:10.1186/s13052-022-01260-3)
Supplement: Supplementary file 2 — Additional file 2: Supplementary file 2. List of participating centres, complete list of participating centres. [file 13052_2022_1260_MOESM2_ESM.docx]

**List of participating centres**

Ospedale Del Ponte; Varese | Ospedale Infermi Rimini; Rimini | Azienda ospedaliera Papardo; Messina | Ospedale San Giovanni di Dio; Agrigento | ASST Lariana; Como | Ospedale San Giovanni di Dio; Crotone | ASST-Bergamoest Ospedale Bolognini; Seriate (BG) | Ospedale San Bortolo di Vicenza; Vicenza | ASST di Lecco; Lecco | Ospedale Guglielmo da Saliceto; Piacenza | Policlinico; Modena | Azienda ospedaliera Sant'Anna e San Sebastiano; Caserta | AAST FBF Sacco Macedonio Melloni; Milano | Ospedale Sant'Anna; Torino | Azienda Ospedaliera Universitaria Integrata; Verona | Fondazione IRCCS Policlinico San Matteo; Pavia | Ospedale Vito Fazzi; Lecce | Policlinico Univ. SS. Annunziata; Chieti | Ospedale Barone Romeo; Patti (ME) | Ospedali Riuniti; Ancona | Presidio Salesi; Ancona | Ospedale Belcolle; Viterbo | Azienda Sanitaria Universitaria del Friuli Centrale; Udine | Ospedale dell'Angelo; Venezia Mestre | Azienda Ospedaliera di Terni; Terni | Ospedale Monaldi; Napoli | San Leonardo ASL Napoli 3 Sud; Castellammare di Stabia (NA) | P.O. G.F. Ingrassia-ASP; Palermo | Humanitas San Pio X; Milano | A.O. San Pio; Benevento | Casa Sollievo della Sofferenza IRCCS; San Giovanni Rotondo (FG) | Presidio Ospedaliero San Francesco; Nuoro | Azienda Ospedaliero Universitaria Pisana; Pisa | Ospedale per l'Emergenza Cannizzaro; Catania | Ospedale Sant’Antonio Abate; Trapani | ASFO; Pordenone | Ospedale Universitario Careggi; Firenze | Policlinico Umberto I, Università La Sapienza; Roma | Villa dei Fiori; Acerra (NA) | Ospedale Antonio Cardarelli; Napoli | Ospedale Di Venere; Bari | Ospedale San Salvatore; L'Aquila | Ospedale San Giovanni Addolorata; Roma | Ospedale Civile Spirito Santo; Pescara | ASST Cremona; Cremona | Policlinico Sant'Orsola-Malpighi; Bologna | Ospedale San Marco; Catania | Azienda Ospedaliera Universitaria di Caglaiari; Cagliari | Ospedale Sant’Anna; Torino | Ospedale Generale di Saronno; Saronno (VA) | Ospedale Maggiore della Carità; Novara | Ospedale Buonconsiglio; Napoli | Ospedale SS Annunziata; Taranto | Ospedale Regionale F.Miulli; Acquaviva delle Fonti (BA) | P.O. Umberto I; Nocera Inferiore (SA) | Ospedale Garibaldi-Nesima; Catania | Ospedale della Versilia - AUSL Toscana Nord Ovest; Lido di Camaiore (LU) | Azienda Ospedaliera Ferrara; Ferrara | ASMN-IRCCS AUSL Reggio Emilia; Reggio Emilia | SS Annunziata; Chieti | Ospedale Maggiore di Lodi; Lodi | Ospedale S. Eugenio; Roma | Ospedale V. Cervello; Palermo | Ospedale Pineta Grande; Castel Volturno (CE) | Ospedale G. Martino; Messina | Ospedale San Pietro FBF; Roma | Clinica Villa dei Platani-Malzoni; Avellino | Policlinico; Bari | Ospedale Ca’ Foncello; Treviso | Ospedale dei Bambini V. Buzzi; Milano | Azienda Ospedaliera Pugliese-Ciaccio; Catanzaro | Ospedali Riuniti; Foggia | Ospedale San Donato; Arezzo | Ospedale Papa Giovanni XXIII; Bergamo | Ospedale S. Maria delle Croci; Ravenna | Ospedale S. Croce; Moncalieri (TO) | GOM Cà Granda Niguarda; Milano | Ospedale Valduce; Como | Grande Ospedale Metropolitano; Reggio Calabria | Policlinico Casilino; Roma | Fondazione IRCCS Cà Granda Ospedale Maggiore Policlinico; Milano | AORN San Giuseppe Moscati; Avellino | Ospedale dei Bambini, ASST Spedali Civili di Brescia; Brescia | Ospedale Evangelico Betania; Napoli | Azienda Ospedaliero-Universitaria; Sassari | Ospedale San Giovanni Calibita Fatebenefratelli; Roma | Presidio Ospedaliero del Levante Ligure; La Spezia | Ospedale dei Bambini Pietro Barilla; Parma | Ospedale San Giovanni di Dio; Firenze | Ospedale A.Perrino; Brindisi |Policlinico Universitario Federico II; Napoli | Ospedale Burlo Garofolo; Trieste | Azienda Ospedaliera Cardinale G. Panico; Tricase (LE) | Ospedale Infantile C. Arrigo; Alessandria | Ospedale Maggiore; Bologna | Ospedale Santa Croce & Carle; Cuneo | Ospedale A.Cardarelli; Campobasso | Ospedale Maria Vittoria; Torino | Azienda Ospedaliera di Cosenza; Cosenza | PO Santa Maria della Speranza; Battipaglia (SA) | Ospedale del Cuore Fondazione Toscana Gabriele Monasterio; Massa | AOR San Carlo; Potenza | Presidio Ospedaliero San Giovanni di Dio; Melfi | Ospedale Giovanni Paolo II; Ragusa | Central Academic Teaching Hospital of Bozen; Bolzano | Ospedale Santa Chiara - APSS; Trento | Ospedale S. Camillo-Forlanini; Roma | P.O. Umberto I; Siracusa | Azienda Ospedale Università di Padova; Padova | Ospedale Santa Maria della Misericordia; Perugia | Ospedale Umberto I; Enna | AOU Vanvitelli; Napoli | Nuovo Ospedale Santo Stefano Prato; Prato | Fondazione Policlinico Universitario Agostino Gemelli IRCCS - Università Cattolica del Sacro Cuore; Roma | Fondazione MBBM Ospedale San Gerardo; Monza | Fondazione Poliambulanza; Brescia | ARNAS Civico Palermo; Palermo | Ospedale San Jacopo; Pistoia | Ospedale di Cavalese; Cavalese (TN) | Ospedale Felice Lotti; Pontedera (PI) | Spedali Riuniti Portoferraio; Portoferraio (LI) | Ospedale Santa Chiara; Trento | Ospedale S. Francesco; Lucca | Spedali Riuniti; Livorno | Ospedale Mugello; Borgo San Lorenzo (FI) | Ospedale di Silandro; Silandro ((BZ) | Ospedale Franz Tappeiner; Merano (BZ) | Ospedale Misericordia; Grosseto | Ospedale San Luca; Lucca | Ospedale di Bressanone; Bressanone (BZ) | Ospedale Santa Maria Annunziata; Bagno a Ripoli-Firenze | Ospedale Santa Maria del Carmine; Rovereto (TN) | Ospedale di Cecina; Cecina (LI) | Ospedale S.M. alla Gruccia; Montevarchi (AR) | Ospedale San Giuseppe; Empoli (FI) | Ospedale San Filippo e Nicola; Avezzano (AQ) | Azienda Sanitaria Provinciale di Vibo Valentia; Vibo Valentia | Ospedale Val Vibrata; Sant'Omero (TE) | Ospedale SS. Annunziata; Sulmona (AQ) | P.O. S. Pio da Pietrelcina; Vasto (CH) | Ospedale San Carlo; Potenza| Ospedale San Giovanni; Lagonegro (PZ) | Ospedale Madonna delle Grazie; Matera | Presidio Ospedaliero di Lamezia Terme; Lamezia Terme | Ospedale di Avezzano; Avezzano | Ospedale Civile di Locri; Locri (RC) | Policlinico Vanvitelli; Napoli | ICM; Agropoli (SA) | Ospedale San Giovanni di Dio; Frattamaggiore (NA) | Ospedale San Paolo; Napoli | Ospedale del Mare; Napoli | Presidio Ospedaliero Agostino Landolfi; Solofra (AV) | Clinica Santa Maria la Bruna; Torre del Greco (NA) | Ospedale di Vaio; Fidenza (PR) | Ospedale Guglielmo da Saliceto; Piacenza | Ospedale Ercole Franchini; Montecchio Emilia (RE) | Ospedale Civile Ramazzini; Carpi (MO) | Ospedale Cesare Magati; Scandiano (RE) | Ospedale Civile di Latisana; Latisana (UD) | Ospedale S. Maria Bianca; Mirandola (MO) | Ospedale Santa Maria delle Croci; Ravenna | Ospedale Santa Maria della Scaletta; Imola (BO) | Ospedale Santissima Annunziata; Cento (FE) | Casa di cura Villa Margherita; Roma | Ospedale San Paolo; Civitavecchia (RM) | Ospedale Cristo Re; Roma | Ospedale Giovan Battista Grassi; Ostia (RM) | Clinica Mater Dei; Roma | Ospedale M.G. Vannini; Roma | Ospedale Dono Svizzero; Formia (LT) | Clinica Santa Famiglia; Roma | Ospedale Provinciale San Camillo de Lellis; Rieti | Ospedale SS. Trinità; Sora (FR) | CDC Clinica Fabia Mater; Roma | Presidio Ospedaliero San Filippo Neri; Roma | Casa di cura Villa Mafalda; Roma | Ospedale Santa Maria Goretti; Latina | Ospedale dei Castelli; Ariccia (RM) | Ospedale Sandro Pertini; Roma | Ospedale Paolo Colombo; Velletri (RM) | Ospedale Santa Scolastica; Cassino (FR) | P.O. Santo Spirito in Saxia; Roma |Ospedale Coniugi Bernardini; Palestrina (RM) | Policlinico San Martino; Genova | Ospedale di Macerata; Macerata | Ospedale Sant'Andrea; La Spezia | Ospedale Evangelico; Genova | Ospedale di Civitanova Marche; Civitanova Marche (MC) | Ospedale Leonardo Felici; Ancona | Ospedale S. Paolo-Santa Corona; Pietra Ligure (SV) | POU ASL3 Ospedale Villa Scassi; Genova | Ospedale Engles Profili; Fabriano (AN) | Ospedale Augusto Murri; Fermo | Ospedale Mazzoni; Ascoli Piceno | Ospedale Madonna del Soccorso; San Benedetto del Tronto (AP) | ASST Mantova; Borgo Mantovano (MN) | Ospedale Cittiglio Verbano; Cittiglio (VA) | Ospedale San Paolo; Milano | ASST Rhodense; Rho (MI) | IRCCS San Raffaele; Milano | ASST Mantova - Presidio di Asola; Asola-Mantova | Ospedale Galmarini; Tradate (VA) | Ospedale di Carate Brianza; Carate Brianza (MB) | Ospedale Desenzano ASST-Garda; Desenzano del Garda (BS) | Ospedale La Memoria ASST-Garda; Gavardo (BS) | ASST-Valle Olona; Fagnano Olona (VA) | Ospedale Civico di Vigevano; Vigevano (PV) | ASST-Franciacorta PO di Iseo; Iseo (BS) | Ospedale Pesenti-Fenaroli; Alzano Lombardo (BG) | Istituto Clinico S. Anna; Brescia | Policlinico San Pietro; Ponte San Pietro (BG) | Ospedale Sacra Famiglia-Fatebenefratelli; Erba (CO) | Ospedale G. Salvini; Garbagnate Milanese (MI) | Ospedale Provinciale di Saronno; Saronno (VA) | Ospedale San L. Mandic Merate-ASST Lecco; Merate (LC) | Ospedale Sant'Antonio Abate; Gallarate (MI) | ASST OVEST MILANESE - H Fornaroli; Magenta (MI) | Ospedale Civile Città di Sesto San Giovanni; Sesto San Giovanni (MI) | Ospedale San Carlo Borromeo; Milano | Ospedale Nuovo di Legnano; Legnano (MI) | Ospedale San Giuseppe; Milano | ASST Valtellina e Alto Lario; Sondrio | ASST Valtellina e Alto Lario; Sondalo (SO) | Ospedale di Vimercate; Vimercate (MB) | Ospedale Civile Manerbio; Manerbio (BS) | ASST Crema-Ospedale Maggiore; Crema (CR) | Presidio di Chiari ASST Franciacorta; Chiari (BS) | Ospedale Santa Maria Stelle; Melzo (MI) | P.O. Desio-ASST Monza; Desio (MB) | Fondazione IRCCS Policlinico San Matteo; Pavia | Ospedale SS. Annunziata; Savigliano (CN) | Ospedale Civile Cirie'; Ciriè (TO) | Ospedale Martini; Torino | Umberto I; Corato (BA) | AOU Maggiore della Carità; Novara | Ospedale S. Andrea; Vercelli | Ospedale Santi Pietro e Paolo; Borgosesia (VC) | Presidio Ospedaliero SS. Trinità; Borgomanero (NO) | Ospedale Lorenzo Bonomo; Andria (BT) | Ospedale di Rivoli; Rivoli (TO) | Ospedale Cardinal Massaia; Asti | Ospedale Castelli; Verbania (VB) | A.O. Mauriziano; Torino | Ospedale S. Giacomo; Monopoli (BA) | Ospedale Mons. AR Dimiccoli Barletta Asl BT; Barletta (BT) | Ospedale della Murgia; Altamura (BA) | Ospedale Santa Caterina Novella; Galatina (LE) | Ospedale Edoardo Agnelli; Pinerolo (TO) | Ospedale Maggiore di Chieri; Chieri (TO) | Ospedale Civico di Chivasso; Chivasso (TO) | Ospedale Santo Spirito; Casale Monferrato (AL) | Ospedale San Giacomo; Novi Ligure (AL) | Policlinico Universitario G. Martino; Messina | P.O. CTO Pediatria-nido; Iglesias (CI) | AOU Policlinico Paolo Giaccone; Palermo | Azienda Ospedaliera Gravina; Caltagirone (CT) | Ospedale Nostra Signora di Bonaria; San Gavino Monreale (VS) | Ospedale Giovanni Paolo II; Olbia (OT) | Clinica Triolo-Zancla; Palermo | Presidio Ospedaliero Duilio Casula, AOU Cagliari Inico Monserrato; Cagliari | Ospedale San Martino; Oristano | AO G. Brotzu; Cagliari | Ospedale Carlo Basilotta; Nicosia (EN) | Ospedale Santa Marta e Santa Venera; Acireale (CT) | Ospedale San Lorenzo; Valdagno (VI) | Ospedale di Monselice; Monselice (PD) | Ospedale Civile di Chioggia; Chioggia (VE)| Ospedale di Conegliano; Conegliano (TV) | Ospedale Vittorio Veneto; Vittorio Veneto (TV) | Ospedale Sant Valentine; Montebelluna (TV) | Ospedale Pederzoli; Peschiera (VE) | Ospedale Civile; San Donà (VE) | Ospedale di Aosta; Aosta | Ospedale San Bassiano; Bassano (VI) | Ospedale di Cittadella; Cittadella (PD) | UOC Pediatria Mirano; Mirano (VE) | Ospedale di Dolo; Dolo (VE) | Ospedale di Rovigo; Rovigo | Ospedale di Adria; Adria (RO) | Ospedale Cazzavillan; Arzignano (VI) | Ospedale Santorso; Santorso (VI) | Ospedale Gubbio-Gualdo Tadino; Gubbio (PG) | Ospedale San Giovanni Battista; Foligno (PG) | Ospedale San Matteo degli Infermi; Spoleto (PG) | Ospedale Oderzo; Oderzo (TV) | Ospedale Generale Mater Salutis; Legnago (VR)
